# Opium in Pregnancy and Parturition

**Published:** 1876

**Authors:** T. Curtis Smith

**Affiliations:** Middleport, Ohio


### SOUTHERN MEDICAL RECORD.

VOL. VI. JANUARY AND FEBRUARY, 1876. NOS. ~~10 & 11~~. 1 & 2.

### ORIGINAL COMMUNICATIONS.

### OPIUM IN PREGNANCY AND PARTURITION.

BY T. CURTIS SMITH, M. D., MIDDLEPORT, OHIO.

The use of opium during the course of gestation is seldom needed, as long as the process continues to be normal, but in many of the accidents and complications that arise, it often becomes indispensable. In many cases of extreme nausea it is valuable, while in some cases it only serves to aggravate the trouble. In accidents that cause threatened abortion or miscarriage, there is probably no single remedy in the Materia Medica as often used to prevent this unhappy result, when not positively contra-indicated by some existing condition, or more than ordinary idiosyncrasy, it forms the sheet anchor among preventives. Even where it does seem contra-indicated by a strong tendency of blood to the head, this may, in a great measure, be overcome by the *use of the lancet*, or by large doses of the bromide of potassium, and where there is a serious idiosyncrasy, the latter remedy will greatly aid in preventing present or sequential effects. Its uses to prevent abortion or premature labor from accidents, are too well known to require further comment at my hands. But in cases where the time for labor is so near at hand that the lady does not know whether her "time is up or not," the judgment of the attendant is necessarily taxed to ascertain whether labor has really begun or whether the pains suffered are not due to some morbid condition of the nervous system, to collected feces in the colon or rectum, or to some trivial accident or excitement not deemed worthy of mention by the lady herself, or possibly to irritability of the uterus or neuralgia. These points must be determined by the condition of the nervous system, the regularity of the bowels, character of the pains, whether any accident or excitement has occurred, or the patient has been performing an unusually severe task of labor, whether the womb and vagina are unusually irritable, or whether the patient is of the neuralgic type and has lately suffered from that disease. Not a little can also be inferred from the condition of the vaginal discharge, for when this is entirely absent at the time, and has been for the day or two past no more than common, it contra-indicates the commencement of labor. The condition of the cervix is also strongly indicative. If it is not yet obliterated, nor dilated, there is little prospect of labor coming on at the time. The pains in many of these cases, even where the os is somewhat dilated, is apt to be of a very grinding, distressing character, and examinations made during the pains fail to show that there is much effect exerted on the os. Under these circumstances a full dose of opium will allay the pains, (for they are more pains than real contractions) quiet the nervous system, or overcome the irritable conditions of the uterus, cause the patient to rest quietly and easily, and give nature time to complete her work, and go on to the allotted time when labor should set in.

False labor pains are not very uncommon, and have sometimes caused serious trouble to the physician, not very infrequently impairing his reputation as an obstetrician. I knew a physician once to remain three days and nights with a lady, who was suffering from pains of this character, (so called) expecting all the while that in a few hours the work would certainly be accomplished. Meanwhile he plied her with hot teas, hot pediluvia, ergot, etc., all to no good effect, and certainly much to the annoyance and detriment of the patient. After the lapse of the three days, the patient and friends became wearied and impatient. Counsel was asked for and obtained. After examining the patient and observing a few pains, the history of the three days' proceedings having been learned, the consulting physician, much to the astonishment of the attendant, gave it as his opinion that the lady was not in labor at all, and the pains were all spurious. A large opiate was given. In a short time the pains ceased, and all went away relieved except the attending physician. Two weeks elapsed before any pain was again noticed by the lady. This time the consulting physician was called, and in a short time natural delivery of a healthy child was effected. It is needless to relate how mortified a man thus defeated for want of a little practical knowledge must feel, or how the lady gossips of the community would rattle away to his detriment as an obstetrician.

Only a short time since I was call to see a lady who was expecting her third confinement. She stated that her time was very near out according to her calculation, and it might be altogether out for all she knew. In making an examination during one of the hardest pains, I discovered that she was very tender to the touch, that there was no vaginal discharge, and from her statement there had been none. The os was a very little dilated, although the neck was not fully obliterated. The pains were of the most distressing, grinding, character. Suspecting spurious labor pains, I enquired if she had not been overdoing, or had met with any accident or unusual excitement. At first she said no, but recalled the fact that two nights previous she had walked the floor with a sick child for several hours, since which time she had not felt as well as usual. This circumstance, together with the want of evidence that labor was normal, at once caused me to diagnose spurious labor pains. A large dose of opium was given at once, to be repeated if needed. After the first dose the trouble vanished and she went on a month before she was again troubled with pains, this time being delivered without trouble, of a fine healthy infant. This was certainly much better than to permit the distress to go on, premature delivery to take place, or like the instance before related, watch the case for days, and then have to wait a fortnight and see the case fall into another accoucheur's hands by virtue of his better practice. Several very similar cases could be related, but as this is a typical one, it will answer for all.

I will, however, refer to a mistake of my own which it will be but fair to relate, as we ought to record our mistakes and failures with our happy hits and successes. Some four years ago I was called to see a lady in confinement. She declared her time was not out. It was her first child, though she had had two abortions before this time. The pains were very severe and of the character described in the preceding cases. The taxis showed that the neck was not yet obliterated, as it was fully a half inch in length, and the os would not admit the tip of the finger. Believing it an instance of threatened premature labor, I ordered four Dover powders of ten grains each. One to be given every two hours till the pains ceased. This was about seven o'clock P.M. I left, stating that I did not think she would be confined for several weeks, or a month. She took two of the powders, when the pains lost their very distressing, grinding character, but became, at the same time, more severe in bearing down. At ten P.M., I was called again by the husband, who stated that the pains were very severe and frequent. On repairing to the house with him, I found the os dilated as large as the palm of the hand, and the uterine contractions forcible and effective. Two hours later she was delivered of a fine, plump ten pound boy. I should have mentioned that at my first call there was a slight vaginal discharge which had come on that afternoon. This should have made me more guarded in my opinion. The facts were, as it seemed to me afterwards, that she was having real labor pains in the first instance, which gave the appearance of being spurious on account of an irritable condition of the womb, which manifestly was present, for when the os was touched in the first examination, she complained bitterly of the distress it caused. The Dover Powders allayed this irritability, and allowed Dame Nature to assume her wonted sway, undisturbed by any irritable womb. But it was the first instance, at that time, where I had seen labor to come on when the neck was not yet obliterated. Since that time I have met with several such instances.

Another case of interest came under my observation in the hands of another practitioner, that greatly annoyed him by its somewhat anomalous course. The lady commenced having uterine pains very severely one morning. He was called, examined, stated all was correct, and sat down to wait, but in two hours the pains ceased. The next three mornings was a repetition of the same phenomena. The friends now asked counsel. The consultant pronounced it a case of uterine neuralgia. Opium was given that morning which stopped the pains. But the next morning they came again. Then, as per agreement with counsel, large doses of quinine were given, and continued to cinchonism, after which she was to have a full dose each night at bed time. Under this treatment the pains failed to return for two weeks, when they came on naturally, and she was properly delivered. In this case opium would relieve the pains but failed to prevent their return.

In many instances where labor has fully begun and the contractions are frequent and forcible, but still are of the most distressing character, and effect nothing or little in the way of advancing the delivery, partly because the os is unyielding, and partly because the pains are far more distressing than expulsive, an opiate, preferably Dover powder, will relieve their distressing character, relax the circular fibres around the os, and permit advancement of the head. It will often assist in relaxing the perineum when rigid.

But opium should not be given in any such cases where there is a marked contra-indication for its use. Often the lancet is indicated in these cases, when opium would prove of no use, or even be detrimental. The judgment of the obstetrician must decide the demands of each individual case. Certainly the vast majority of cases need nothing but to be left alone. Nature, as a rule, does her part well. It is only our business to assist, not to thwart her efforts. I am aware that some practitioners claim an oxytocic property for opium. Such claim cannot well be established. That the uterine contractions sometimes became more severe under its effects I am aware, but where it seems to produce such an effect, it does so only by removing some morbid condition of the uterus, or system that has retarded labor. If it does possess oxytocic properties, why give it to prevent abortion and premature labor? For certainly it would increase rather than abate the trouble. Opium, as far as I have been able to judge, will produce but little effect over natural uterine contractions. It will not sensibly diminish or increase them. But in many a case where I have been in doubt as to whether the contractions were normal and would go on to the completion of delivery, have given a dose of opium, or pulv. Doveri, as a test of their character. In these cases I have found many times that the pains would become more effective and steady, losing any morbid action they had manifested, while in other cases, not apparently dissimilar, the contractions had ceased for days, to come on again normally, and quickly effect delivery.

Doubtless, every practitioner is aware of the great value of opiates, after delivery is effected, in relieving afterpains where these are too distressing to be endured. The fear of their checking the lochial discharge is not well founded, and no woman should be allowed to suffer extremely from these pains, when an agent is at command to check them enough to bring them within the point of endurance.

With all that I have said in favor of opium, I do not wish to be understood as advocating its indiscriminate use during gestation or labor. On the contrary, it requires experience and judgment to decide when to give, and when not to give it. If I have added a mite to enable any to decide these points, my object shall have been accomplished.

